# Hyperspectral Image Classification with Optimized Compressed Synergic Deep Convolution Neural Network with Aquila Optimization

**DOI:** 10.1155/2022/6781740

**Published:** 2022-07-07

**Authors:** Tatireddy Subba Reddy, Jonnadula Harikiran, Murali Krishna Enduri, Koduru Hajarathaiah, Sultan Almakdi, Mohammed Alshehri, Quadri Noorulhasan Naveed, Md Habibur Rahman

**Affiliations:** ^1^Computer Science and Engineering, B V Raju Institute of Technology, Narsapur, Medak, Telangana, India Pin: 502313; ^2^School of CSE, VIT-AP University, Vijayawada, Pin: 522237, Andhrapradesh, India; ^3^Computer Science and Engineering, SRM University-AP, Amaravati, India; ^4^Department of Computer Science, College of Computer Science and Information System, Najran University, Najran, Saudi Arabia; ^5^Department of Computer Science, College of Computer Science, King Khalid University, Abha, Saudi Arabia; ^6^Dept. of Computer Science and Engineering, Faculty of Engineering and Technology, Islamic University, Kushtia-7003, Bangladesh

## Abstract

The classification technology of hyperspectral images (HSI) consists of many contiguous spectral bands that are often utilized for a various Earth observation activities, such as surveillance, detection, and identification. The incorporation of both spectral and spatial characteristics is necessary for improved classification accuracy. In the classification of hyperspectral images, deep learning has gained significant traction. This research analyzes how to accurately classify new HSI from limited samples with labels. A novel deep-learning-based categorization based on feature extraction and classification is designed for this purpose. Initial extraction of spectral and spatial information is followed by spectral and spatial information integration to generate fused features. The classification challenge is completed using a compressed synergic deep convolution neural network with Aquila optimization (CSDCNN-AO) model constructed by utilising a novel optimization technique known as the Aquila Optimizer (AO). The HSI, the Kennedy Space Center (KSC), the Indian Pines (IP) dataset, the Houston U (HU) dataset, and the Salinas Scene (SS) dataset are used for experiment assessment. The sequence testing on these four HSI-classified datasets demonstrate that our innovative framework outperforms the conventional technique on common evaluation measures such as average accuracy (AA), overall accuracy (OA), and Kappa coefficient (k). In addition, it significantly reduces training time and computational cost, resulting in enhanced training stability, maximum performance, and remarkable training accuracy.

## 1. Introduction

Due to the fast growth of photonics with optics, sensors in hyperspectral (HS) are needed to install in several satellites. HSI classification is an essential and challenging task that is targeted towards labelling each pixel contained in a hyperspectral image. HSI images contained spatial-spectral information which is useful for detecting scene objects [1]. This had been used in many fields like environmental surveillance, astronomy, and precise agriculture [2].

In the earlier days, HSI classification was done by the machine learning methods such as support vector machines (SVM) [3, 4], k-nearest neighbor (KNN) [5, 6], multinomial logistic regression (MLR) [7, 8], and decision tree [9, 10]. Within the similar data which exists, spectral changes in various materials and various spaces might have the same features, so the attained details were still corrupt because of inadequate spatial structure feature extraction. To solve these issues, it is hard to perfect the classification of HSI. So, numerous spectral and spatial feature extraction methods are proposed.

These techniques have validated major classification performance, which is not in effect for classifying HSI in difficult situations. In recent times, deep learning techniques had achieved maximum success for this kind of task [11–13]. So, this method had reached admirable performance for different analysis-oriented tasks, e.g., object recognition and image classification. To classify HSI, entire spatial and spectral perspectives must be considered for the processing. Intuitively, HSI consists of a higher number of images and every image signifies electromagnetic spectrum classification. Temporarily, the spatial perspective denotes 2D spatial data of objects consistent in the HSI. Thus, HSI is typically denoted as the 3D spectral-spatial data. Therefore, many methods had been proposed in the literature [14, 15].

Towards concurrently modelling spectral-spatial data, certain developer attempts were made. This method performed operations in a stacked manner along with convolution over spectral and spatial feature space in a stacked manner, named CNN model [16]. Apparently, the benefit of this CNN model may create rich feature maps. Moreover, the major drawback of this method is threefold. Initially, It is hard to generate a deeper CNN structure. An intention in the resultant area increasingly improves through cumulative amount in the convoluted function that confines the interpretation ability and depth of the model. Next to that, the cost of the memory is too expensive while maximum convolution operations were performed [17–20]. To reduce the abovementioned challenges, we introduced the new CNN model namely compressed synergic deep convolution neural network with Aquila optimization (CSDCNN-AO).

The significant goals to achieve the above-said objectives are listed below:

- to determine the suitable deep learning method which provides huge support for HSI image classification.
- To reduce the complexity and loss function in classification.
- To develop the future outcome based on both present and traditional output.

The major contribution of this technique is given below.

This combination will reduce the learning complexity of the wavelet concept and reduce the loss function with the Aquila optimization. This Aquila optimization method could reduce the enormous amount of data features by maintaining its unique possessions and using less time for computation and less memory space. Furthermore, a synergic deep convolutional neural network (CNN) is useful and intended for getting an initial result, similarly, the CNN weights are optimized by Aquila optimization for reducing an error rate. Here, the key role is the compression of data with the Aquila optimization technique with CNN for increasing accuracy with maximum steadiness among both exploitation and exploration of optimization.

The organization of the work is given below:

the literature survey is given in Section 2. In Section 3, the proposed methodology is given. In Section 4, the experimental results and discussions are explained. At last, in Section 5, the conclusion is given.

## 2. Literature Review

Yang et al. [21] present a novel synergistic CNN for an accurate HSI classification. The SyCNN contains the hybrid structure of 2D and 3D CNNs with a data interaction module with feature learning that fuses both spatial and spectral HSI data. Moreover, it presents a three-dimensional process earlier to a fully connected layer that supports and extracts features effectively. But still, they could not handle high-dimensional data.

Li et al. [22] suggested an HSI model called local and hybrid dilated convolution fusion network (LDFN) that combines both the local and rich spatial features through expanding the perception field. Initially, several functions were considered, such as dropout, standard convolution, batch normalization, and average pooling. After that, both local and dilated convolution operations were involved in efficient spatial-spectral feature extraction. On the other hand, parameters were manually selected in the suggested paper.

Patel et al. [23] suggested HSI categorization by an autoencoder through CNN (AECNN). Pre-processed by autoencoder-enhanced HSI features that helped towards obtaining optimized weights in CNN initial layers. Thus, here, CNN with a shallow model could be applied towards extracted features from the HSI data. But still, they need to cover more contextual information and advanced strategies for robustification of the spatial information.

Wang et al. [24] suggested a semi-supervised HSI classification model which improved deep learning. Here, the suggested model namely the arbitrary multiple graphs method, and then replaced skilled learning with the anchor graph method that could be labelled a significant unlabelled data automatically and precisely. In this, the number of training samples is limited.

Shi et al. [25] presented a model namely the 3D coordination attention mechanism (3DCAM). This attention process could not attain the HIS's spatial position in both vertical and horizontal ways. Also, HSIs spatial and spectral data were extracted, using CNN. The drawback is that the implementation complexity is not considered.

Zhao et al. [26] suggested combining stacked autoencoder (SAE) with 3D deep residual network (3DDRN) to classify HSI. An SAE neural network was designed to reduce HSI size. 3DCNN and residual network module were used to develop 3DDRN. The 3DDRN extracted spectral-spatial features from dimension-reduced 3D HSI cubes. 3DDRN continuously identified deep features, which were passed into SoftMax to complete classification. Batch normalization (BN) and dropout were used to avoid overfitting training data.

Yin et al [27] developed a spatial-spectral mixed network for HSI categorization. The network collects spatial-spectral information from HSI using three layers of 3-D convolution and one layer of 2-D convolution. This network employs Bi-LSTM to boost spectral band interactions and extract spectral features as a series of images. Combining two FC layers and utilising SoftMax for classification creates a unified neural network. However, the model misclassified samples in the dataset.

Paul et al. [28] developed SSNET, which blends 3D and 2D convolutions of HSI spectral-spatial information with SPP for creating spatial features at various scales. SPP is employed in two-dimensional local convolutional filters for HSI classification because it resists object distortions. SPP layer's fixed feature vector output reduces trainable parameters and improves classification performance. They do, however, have a complicated structure.

Zhang et al. [29] introduced an SSAF-DCR for hyperspectral image classification. Three components were linked to extract features in the recommended network. First, a dense spectral block reuses spectral characteristics as much as possible. Then, a spectral attention block refines and optimises the spectral features. In the second segment, a dense spatial block and an attention block pick spatial features. But in this, the selection of the number of features is not considered.

Yan et al. [30] offer a 3D cascaded spectral-spatial element attention network (3D-CSSEAN) for picture classification. Using the spectral element attention module and the spatial element attention module, the network may concentrate on key spectral and spatial aspects. Two-element attention modules were built using activation functions and element-wise multiplication. The model can extract classification-helping properties and is computationally efficient. The network structure is also suitable for small sample learning since the attention module has few training parameters. On the other hand, obtaining labelled samples are expensive and difficult.

To overcome existing challenges, our proposed work introduces novel techniques which are discussed in the following section.

## 3. Proposed Synergic Deep Learning Model

**Figure 1:**
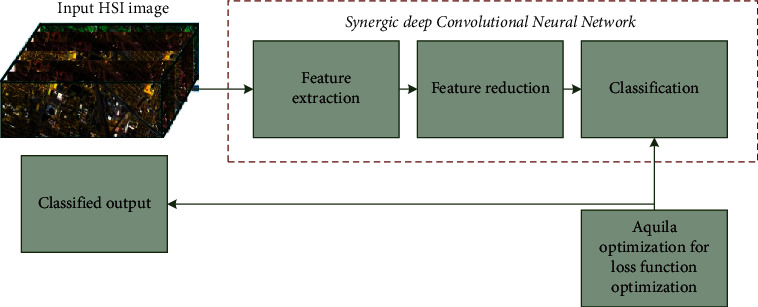
Schematic representation of the proposed methodology.

Let us assign the hyperspectral image *x*=[*X*_1_, *X*_2_, *X*_3_,…,*X*_*s*_]^*t*^ ∈ *r*^*s*×(*c* × *d*)^, where *s* represented entire bands with *c* × *d* band samples. Additionally, *t* is the sample in which *x*=(*X*_*i*_, *Y*_*i*_) ∈ (*r*^*s*×(*c* × *d*)^, *r*^*y*^) with *Y*_*i*_ labels. Usually, HSI classification is affected due to inter-class similarity and high intra-class variability. To compensate for these issues, we introduce the proposed technique namely, the synergic deep learning model with the feature reduction principle. This method minimizes complexities for computation by reducing spectral and spatial feature dimensions. Here, we evaluate the efficiency of the subsequent feature suppression methods using a hybrid synergic deep CNN model. The proposed synergic deep learning model consists of synergic deep learning (SDL)-based feature extraction, feature reduction, classification, and loss function optimization. The schematic representation of the proposed method is represented in Figure 1, which is given in the following sections.

### 3.1. Synergic Deep Convolutional Neural Network Feature Extraction

**Figure 2:**
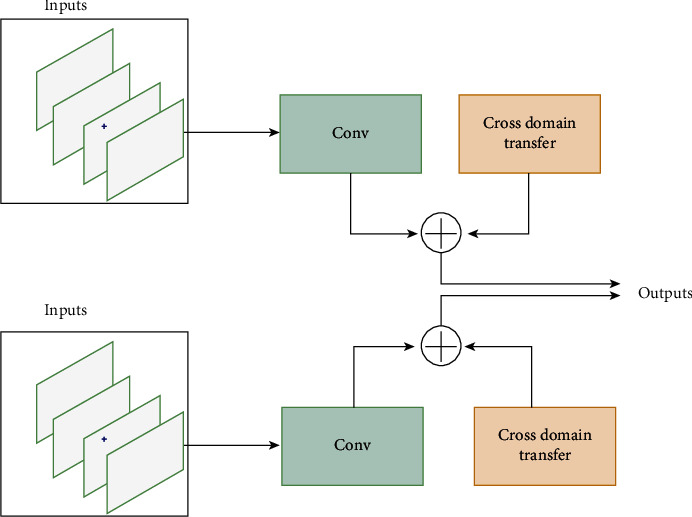
Synergic deep learning model.

In this proposed model as shown in Figure 2, we extract the HSI useful features which are normally represented by the input layer, *n* DCNN components and synergic network (*c*_*n*_^2^). Recently, DCNN yields more attention for the classification which is proposed to reduce the number of input variables and develop the neural network architecture. DCNN is a combination of layers where each layer performs different functions. Pre-processing, convolution, pooling, and final classification operations are sequentially performed in synergic DCNN [31]. The forward process is a convolution operation on the inputs. The multiplication between weights and inputs is combined across layers. The filter has the same number of layers as input volume channels, and output volume has the same depth as the number of filters. In the convolution process, several computations are carried out. Every layer is composed of neurons that take input values, perform calculations, and produces output values, which are forwarded to the next layer. Under CNN, there are four important operations performed in feature learning: the convolution, the activation, the pooling, and the normalization. Before convolution operation, pre-processing is worked out.

#### 3.1.1. Pair Input Layer

Synergic pair input layers are trained randomly, and here, each 200-data group with corresponding class labels is given to the DCNN units. Here, the image is in the size of 224 × 224 × 3. Before applying the data to the next layer, we have to apply the feature reduction principle.

#### 3.1.2. Feature Reduction by Wavelet Transform

In this feature reduction concept, we used wavelet transform with the Haar basis model so that they can handle the high-dimensional data efficiently. Here, two filters *h* and *g* are applied for effective feature reduction. These filters are incorporated with the transforms to yield deducted input coefficients. The following equation is for the feature reduction which is given in equation (1).

(1) $$\begin{array}{c} x^{\prime }=\left[\begin{array}{c} h\\ g \end{array}\right]x{\left[\frac{h}{g}\right]}^{T}=\left[\begin{array}{c} hx\\ gx \end{array}\right]\left[h^{T}g^{T}\right]\\ =\left[\begin{array}{c} hxh^{T}hxg^{T}\\ gxh^{T}gxg^{T} \end{array}\right]. \end{array}$$

As a result of this transformation into the DCNN, learning complexity and learning time can be reduced. In this process, it reduces CNN architecture with the number of features.

#### 3.1.3. DCNN Component

In every DCNN component, we initiate with ResNet-101 architecture which is denoted as DCNN-n (*n* = 1, 2,…, N). This type of architecture is suitable for synergic deep learning (SDL) method. Here, we consider the data sequence with compressed features *x*′={*X*′^(1)^, *X*′^(2)^,…, *X*′^(*n*)^} and output class label series *y*′={*Y*′^(1)^, *Y*′^(2)^,…*Y*′^(*n*)^}. This has to be intended with the *θ* variable which undertakes cross-entropy loss expressed in equation (2).

(2) $$\begin{array}{c} \log\left(\theta \right)=-\frac{1}{n}\left[\sum_{i=1}^{n}\sum_{j=1}^{k}1\left\{Y^{\left(1\right)}=j\right\}\log\frac{e^{z_{j}^{\left(i\right)}}}{\sum_{L=1}^{k}z_{L}^{\left(i\right)}}\right]. \end{array}$$

The above equation (2), *z*^(*i*)^=*f*(*X*^(*A*)^, *θ*) means the forward computing process. In the same way, the variable used in DCNN-n is mentioned as *θ*^*i*^, and these components will not share enormous DCNN components.

In this SDN model, synergic labels in DCNN are applied to input layers, embedding, and learning layers. In SDN, the consequence data pair is denoted as (*z*_*I*_, *z*_*J*_), and this pair of input is given to (DCNNi, DCNNj). Output from the FC layer is given in the following equations (3) and (4).

(3) $$\begin{array}{c} f_{I}=\psi \left(z_{I},\theta^{\left(i\right)}\right), \end{array}$$

(4) $$\begin{array}{c} f_{J}=\psi \left(z_{J},\theta^{\left(j\right)}\right). \end{array}$$

In the next stage, all the deep features are embedded *f*_*I*∘*J*_ and the resultant outcome is expressed in the following equation (5).

(5) $$\begin{array}{c} Y_{\text{SDL}}\left(z_{I},z_{J}\right)=\left\{\begin{array}{cc} 1, & \text{if }Y_{I}=Y_{J},\\ , & \text{if }Y_{I}\neq Y_{J}. \end{array}\right. \end{array}$$

Loss in binary cross-entropy is given as below:

(6) $$\begin{array}{c} L^{\text{SDL}}\left(\theta^{\text{SDL}}\right)=Y_{\text{SDL}}\log\;{\overset{⌢}{Y}}_{\text{SDL}}+\left(1-Y_{\text{SDL}}\right)\log\left(1-{\overset{⌢}{Y}}_{\text{SDL}}\right). \end{array}$$

The above expression *θ*^SDL^ represents the synergic attributes, and *Y*_SDL_ represents the synergic forward computation. This process validates data pair classes and yields a recovery response belonging to the synergic (SN) errors.

#### 3.1.4. Training and Testing

In this stage, we do the SN maximization process

(7) $$\begin{array}{c} \left\{\begin{array}{c} \theta^{\left(i\right)}\left(z+1\right)=\theta^{\left(a\right)}\left(z\right)-\gamma \left(z\right).\Delta^{\left(i\right)},\\ \theta^{\text{SDL}\left(i\right)}\left(z+1\right)=\theta^{\text{SDL}\left(a\right)}\left(z\right)-\gamma \left(z\right).\Delta^{\text{SDL}\left(i,j\right)}, \end{array}\right. \end{array}$$

where, SDL(*i*, *j*) and *γ*(*z*) represents the learning rate

(8) $$\begin{array}{c} \Delta^{\left(i\right)}=\frac{\partial L^{\left(a\right)}\left(\theta^{\left(i,j\right)}\right)}{\partial \theta^{\left(i,j\right)}}+\vartheta \sum_{j=1,j\neq a}\frac{\partial L^{S\;DL\left(i\right)}\left(\theta^{S\;DL\left(i,j\right)}\right)}{\partial \theta^{S\;DL\left(i,j\right)}},\\ \Delta^{S\;DL\left(i\right)}=\frac{\partial L^{S\;DL\left(a\right)}\left(\theta^{S\;DL\left(i,j\right)}\right)}{\partial \theta^{S\;DL\left(i,j\right)}}, \end{array}$$

where, *ϑ* refers to the trade-off among synergic error and classification sub-model. Additionally, test data classification belonging to the SN DCNN component is processed under some of the prediction vectors which are represented as *p*^(*i*)^=(*P*_1_^(*i*)^, *P*_2_^(*i*)^,…, *P*_*k*_^(*i*)^). Further, the test data class label is deliberated as below:

(9) $$\begin{array}{c} y{}^{\prime }\left(z\right)=\underset{\upsilon }{\arg\max}\left\{\sum_{U-1}^{K}P_{1}^{\left(U\right)},\ldots ,\sum_{U-1}^{K}P_{\upsilon }^{\left(U\right)},\ldots ,\sum_{U-1}^{K}P_{k}^{\left(U\right)}\right.. \end{array}$$

### 3.2. Image Classification

This is the final stage to classify the HSI images concerning the different class labels. This classification is performed under the SoftMax layer which has more attention for the multi-label classification. It leads to a mapping function on behalf of the *C* input vector as of space *n* to class *k* labels, which is given in equation (10).

(10) $$\begin{array}{c} \upsilon_{Q}=\frac{e\left(\theta_{zQ}C\right)}{\sum_{k=1}^{k}e\left(\theta_{K}^{z}C\right)}, \end{array}$$

where, *Q*=1,2,…, *k* and *θ*_*k*_=[*θ*_*K*1_, *θ*_*K*2_ … *θ*_*Kn*_]^*z*^ refers the weights, and this has to be tuned using the optimization process. As a result, we can reduce the loss function in this architecture.

### 3.3. Loss Reduction by Aquila Optimization Algorithm

Losses in this SDL are reduced by the Aquila optimization algorithm with the weight tuning process. This Aquila optimization algorithm yields the best solution despite the definite limitations.

The mathematical model of Aquila optimization (AO) [32] consists following stages: expanded exploration, narrowed exploration, expanded exploitation, and narrowed exploitation.

#### 3.3.1. Expanded Exploration

In this work, Aquila recognizes the best weight *θ*_*k*_ based on the best hunting area. Here, the best hunting area refers to the minimum losses. In this process, the AO (weight optimization) extensively explores extraordinary soar to conclude the search space area.

(11) $$\begin{array}{c} \theta_{1}\left(T+1\right)=\theta_{\text{BEST}}\left(T\right)\times \left(1-\frac{T}{t}\right)+\left(\theta_{m}\left(T\right)-\theta_{\text{BEST}}\left(T\right)*RAN\;D\right), \end{array}$$

where, *θ*_1_(*T*+1) refers to the next iteration solution, and this is estimated by the initial search method *θ*_1_. *θ*_BEST_(*T*) is considered as the best until iteration T. Expanded search (exploration) is controlled by the (1 − *T*/*t*) iteration. In addition to that, *θ*_*m*_(*T*) represented the current location mean value which is calculated in the following equation. *t* and *T* are the maxima and current iterations.

(12) $$\begin{array}{c} \theta_{m}\left(T\right)=\frac{1}{n}\sum_{a=1}^{n}\theta_{a}\left(T\right), \end{array}$$

where, *n* is the population size.

#### 3.3.2. Narrowed Exploration

In this stage, AO barely discovers (explores) the certain space of the targeted prey for the solution.

(13) $$\begin{array}{c} \theta_{2}\left(T+1\right)=\theta_{\text{BEST}}\left(T\right)\times \text{LEVY}\left(d\right)+\theta_{r}\left(T\right)+\left(v-u\right)*\text{RAND}, \end{array}$$

where, *θ*_2_(*T*+1) is the next iteration solution. LEVY(*d*) and *d* is the levy flight distribution function and dimension space, respectively. Additionally, *θ*_*r*_(*T*) is the random solution which is taken from the range of (1,…, *n*).

(14) $$\begin{array}{c} \text{LEVY}\left(\text{d}\right)=S\times \frac{U\times \rho }{{\left|V\right|}^{1/\beta }}, \end{array}$$

where, *S* refers to the constant which has the value of 0.01. Moreover, *U* and *V* are constant numbers.

(15) $$\begin{array}{c} \rho =\frac{\Gamma \left(1+\beta \right)\times \;\sin\;e\left(\pi \beta /2\right)}{\Gamma \left(1+\beta /2\right)\times \beta \times 2\left(\beta -1/2\right)}. \end{array}$$

In the above equation (15), *β* is the constant value. Moreover, the value of *u* and *v* are calculated as follows, which is used for spiral search in this optimization.

(16) $$\begin{array}{c} v=R\;\cos\left(\phi \right),\\ u=R\;\sin\left(\phi \right),\\ R=R_{1}+\varepsilon \times d_{1},\\ \phi =-\varpi \times d_{1}+\phi_{1}, \end{array}$$

*R*_1_ has the values from 20 to toward fixed search cycles, and *ε* has the value of 0.00565. *d*_1_ differs based on dimension, then *ϖ* is a minimum value which is a constant 0.005.

#### 3.3.3. Expanded Exploitation (X3)

In this stage, weight optimization exploits the accurate value of the solution for getting nearer to prey and attack.

(17) $$\begin{array}{c} \theta_{3}\left(t+1\right)=\theta_{\text{BEST}}\left(T\right)-\theta_{m}\left(T\right)\times \delta -\text{RAND}+\left(\left(B_{\text{upper}}-B_{\text{lower}}\right)\times \text{RAND}+B_{\text{lower}}\right)\times \alpha , \end{array}$$

where *θ*_3_(*t*+1) refers to the next iteration solution, and *θ*_BEST_(*T*) represents the estimated prey location. In addition to that, *θ*_*m*_(*T*) represents the current mean value at the *T*th iteration, and RAND means the random value which is between 0 and 1. *α* and *δ* are the small values (0, 1) which are adjustment parameters for the exploitation process. *B*_upper_ and *B*_lower_ represents the upper and lower bound of the problem, respectively.

#### 3.3.4. Narrowed Exploitation

In this phase, attacking is processed in the last location.

(18) $$\begin{array}{c} \theta_{4}\left(T+1\right)=qf\times \theta_{\text{BEST}}\left(T\right)-\left(g_{1}\times \theta \left(T\right)\times \text{RAND}\right)-g_{2}\times \text{LEVY}\left(d\right)+\text{RAND}\times g_{1}, \end{array}$$

where *θ*_4_(*T*+1) demonstrates the next iteration solution. *qf* mentions the quality function which is applied for balancing the search strategies. *g*_1_ specifies several optimization motions that are applied for tracking the prey. *g*_2_ specifies the values that are reduced from two to zero. *θ*(*T*) represents *t* iteration with the current solution.

(19) $$\begin{array}{c} qf\left(T\right)=T^{2\times \text{RAND}\left(\right)-1/{\left(1-t\right)}^{2}},\\ g_{1}=2\times \text{RAND}\left(\right)-1,\\ g_{2}=2\times \left(1-\frac{T}{t}\right). \end{array}$$

*qf*(*T*) refers to the *t*th iteration's quality function, and RAND means random value between 0 and 1. *T* and *t* presents the maximum and current iteration, respectively. Levy(D) is the levy flight distribution function calculated using equation (6). As a result, we can get optimum weights which reduces losses in the architecture.

## 4. Experimental Results and Discussion

In our work, we have used four HSI datasets which are used for analyzing our proposed CSDCNN-AO technique. Here, we use Houston U (HU) dataset [33], Indiana Pines (IP) [34], Kennedy Space Center (KSC) [35], and Salinas Scene (SS) dataset [17]. In the case of the IP dataset, the size of the dataset is 145 × 145. For the KSC dataset, the size is equivalent to 512 × 614 with 13 classes of ground truths.

### 4.1. Dataset and Its Description

#### 4.1.1. Houston U (HU) Dataset

The first dataset is GRSS DFC 2013, which measures 349 1905 bytes, and has 144 bands spanning the wavelength range 380–1050 nm. It was obtained by the National Center for Airborne Laser Mapping (NCALM) and has a spatial resolution of 2.5 metres over the University of Houston. The picture is separated into two halves: the bright and dark sections. The bright section has 4143 samples, whereas the dark section contains 824 samples.

#### 4.1.2. Indiana Pines (IP)

This agricultural dataset was collected in 1992 from Northwest Indiana utilising the Airborne Visible/Infrared Imaging Spectrometer (AVIRIS) sensor. It has 145 × 145 pixels and 16 vegetation classifications with 20 m per pixel spatial resolution. After removing 4 zero bands and 20 bands affected by water absorption effects, 200 spectral bands ranging from 400 to 2500 nm with 10-nm intervals were used for analysis.

#### 4.1.3. Kennedy Space Center (KSC)

The AVIRIS instrument in Florida collected the Kennedy Space Center dataset in 1996. It has a resolution of 512 by 614 pixels, 176 bands, and 13 categories.

#### 4.1.4. Salinas Scene (SS) Dataset

Experiments on the Salinas Scene collected by the AVIRIS sensor over Salinas Valley, California, USA, with a spatial resolution of 3.7 m per pixel in the wavelength range of 0.4–2.5 m and a spectral resolution of 10 nm, used a second set of AVIRIS data. It measures 512 × 217 × 224 pixels (water absorption bands included).

The model for comparison enactment depending on the IP dataset through different classes are evaluated.

In this Table 1, we evaluated the classification performance for the Indian Pines Scene dataset. Here, overall accuracy, average accuracy, and Kappa coefficients are evaluated. From the results, we can show that our proposed CSDCNN-AO yields maximum performance than other techniques. In Table 1, CSDCNN-AO achieves a better result for the 13th class. In the case of CSDCNN, the 8th class achieves a better performance. For SDCNN, the 16th class has the maximum performance. DCNN also attains maximum performance for the 16th class only. For RNN, it has the maximum performance under the 6th class.

**Figure 3:**
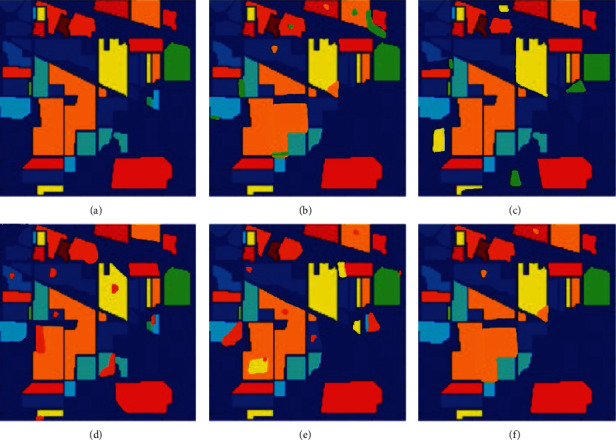
HSI classified image for IP dataset (a) original ground truth image (b) CSDCNN-ALO, (c) CSDCNN-PSO, (d) CSDCNN-WOA, and (e) CSDCNN-GWO (f) CSDCNN-AO.

In the above Figure 3, (a) represents the original image and here we evaluated the results of the proposed algorithm with other algorithms like CSDCNN-ALO [36], CSDCNN-PSO [37], CSDCNN-WOA [38], and CSDCNN-GWO [39]. Different application [40–45] were used in different fields for optimization. Among these methods, our proposed work yields the maximum performance since the performance of our proposed work is nearly equivalent to the original ground truth image compared to others.

In this Table 2, we evaluated the classification performance for the KSC dataset. Here, abovementioned performances are evaluated. From the results, we can show that our proposed CSDCNN-AO yields the maximum performance than other techniques. In Table 2, CSDCNN-AO achieves a better result for the 10th class. In the case of CSDCNN, the 11th class achieves a better performance. For SDCNN, the 13th class has the maximum performance. DCNN attains the maximum performance for the 8th class. For RNN, it has the maximum performance under the 6th class.

**Figure 4:**
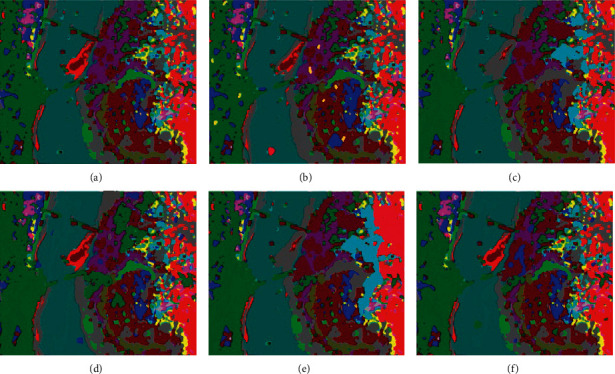
HSI classified image for KSC dataset: (a) original ground truth image, (b) CSDCNN-ALO, (c) CSDCNN-PSO, (d) CSDCNN-WOA, (e) CSDCNN-GWO, and (f) CSDCNN-AO.

In the above Figure 4, (a) represents the original image and here we evaluated the results of the proposed algorithm with other algorithms like CSDCNN-ALO, CSDCNN-PSO, CSDCNN-WOA, and CSDCNN-GWO. From these methods, our proposed work yields the maximum performance since the obtained proposed image is nearly equivalent to the original ground truth image.

In this Table 3, we evaluated the classification performance for the Salinas Scene (SS) dataset. From the results, we can show that our proposed CSDCNN-AO yields the maximum performance than the other techniques. In Table 3, CSDCNN-AO achieves a better result for the 13th class. In the case of CSDCNN, the 16th class achieves a better performance. For SDCNN, the 14th class has the maximum performance. DCNN also attains the maximum performance for the 16th class only. For RNN, it has the maximum performance under 11th class.

**Figure 5:**
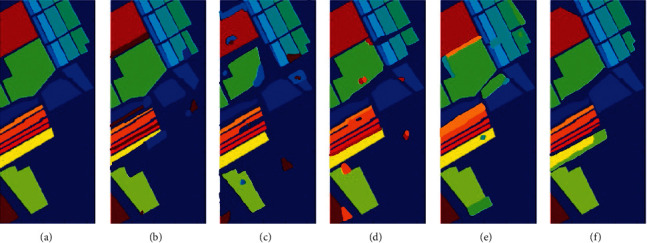
HSI classified image for SS dataset (a) original ground truth image (b) CSDCNN-ALO, (c) CSDCNN-PSO, (d) CSDCNN-WOA, and (e) CSDCNN-GWO (f) CSDCNN-AO.

In the above Figure 5, (a) represents the original image and here we evaluated the results of the proposed algorithm with other algorithms like CSDCNN-ALO, CSDCNN-PSO, CSDCNN-WOA, and CSDCNN-GWO. From these methods, our proposed work yields the maximum performance since the obtained proposed image is nearly equivalent to the original ground truth image.

In Table 4, we evaluate the classification performance for the Houston U dataset. From the results, we can show that our proposed CSDCNN-AO yields the maximum performance than the other techniques. In Table 4, CSDCNN-AO achieves a better result for the 11th class. In the case of CSDCNN, the 8th class achieves a better performance. For SDCNN, the 15th class has the maximum performance. DCNN also attains the maximum performance for the 14th class only. For RNN, it has the maximum performance under 8th class.

**Figure 6:**
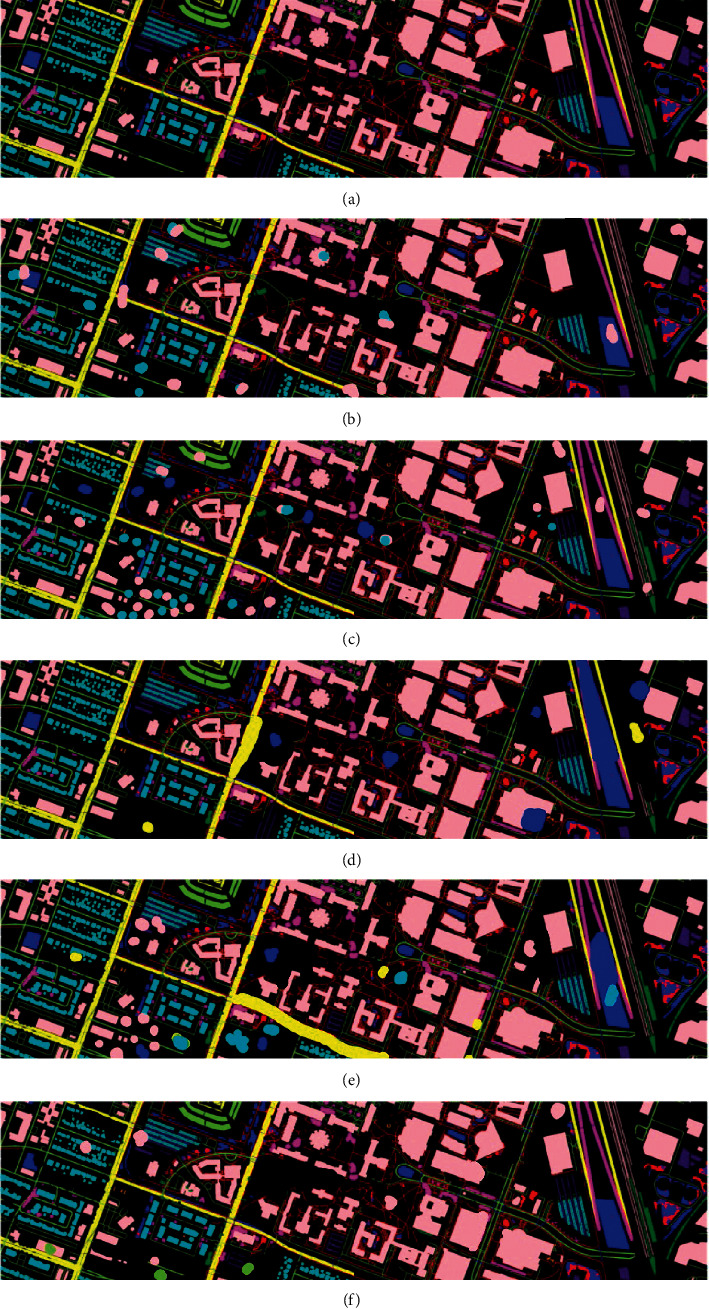
HSI classified image for Houston U dataset: (a) original ground truth image, (b) CSDCNN-ALO, (c) CSDCNN-PSO, (d) CSDCNN-WOA, (e) CSDCNN-GWO, and (f) CSDCNN-AO.

In the above Figure 6, (a) represents the original image then we evaluated the outcome of the proposed algorithm with other algorithms like CSDCNN-ALO, CSDCNN-PSO, CSDCNN-WOA, and CSDCNN-GWO. From these methods, our proposed work yields the maximum performance since the obtained proposed image is nearly equivalent to the original ground truth image.

The input images were obtained from the four datasets. Results are obtained after feature extraction, feature reduction, classification, and loss function optimization. The four different datasets taken for testing purposes are the HU dataset, IP, KSC, and SS dataset. These four datasets have shown promising results in this classification. The results (i.e., computational complexity, overall accuracy and loss functions) that are obtained by these datasets are given in the following figure.

**Figure 7:**
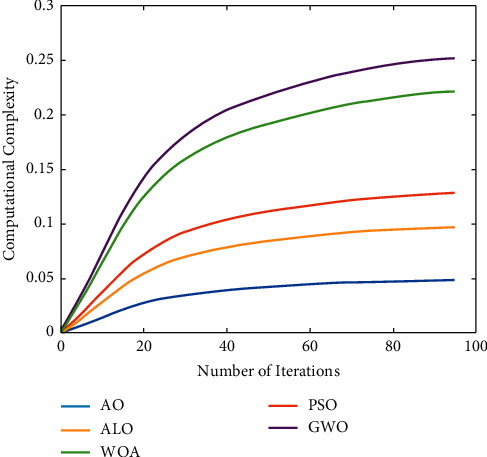
Comparative analysis of computational complexity.

The computational complexity attained for various iterations are shown in Figure 7. The usage of various optimizations along with the synergic deep CNN has improved the performance of the proposed algorithm. The computational complexity attained by Aquila optimization is much better as it has identified the optimal solution in lesser number of iterations, due to this the computational complexity has to be increased while increasing the iterations. Not like other meta-heuristic algorithms, this optimization algorithm has provided satisfactory results on weight parameter selection compared to ALO, WOA, PSO, and GWO. Therefore, in this proposed process, the Aquila optimization is encouraged.

**Figure 8:**
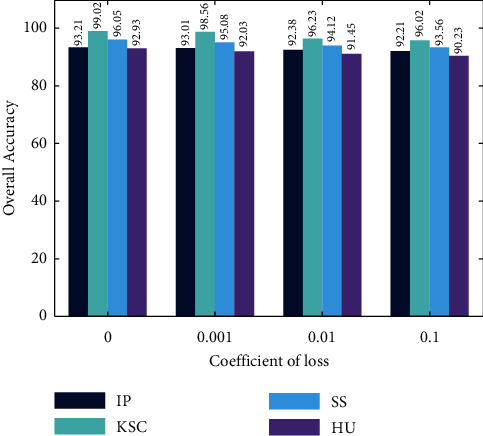
A comparative analysis of overall accuracy.

The overall accuracy comparison for the abovementioned data sets is shown in Figure 8. Among all the datasets, the dataset named KSC has shown a higher accuracy value than other algorithms. These four efficient datasets are taken for comparison. However, the overall accuracy is evaluated with the coefficient loss. The comparison analysis in terms of overall accuracy is affected while coefficient loss is increased. Our proposed work yields maximum accuracy of 99.02%, and this is lagged for the increasing coefficient losses.

**Figure 9:**
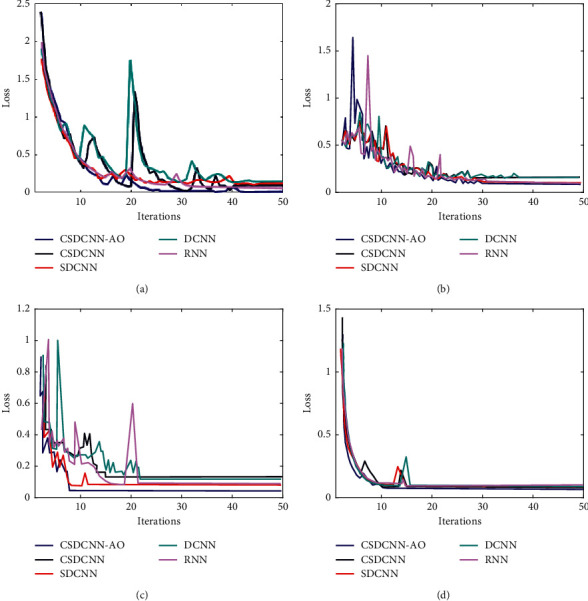
Loss comparison for (a) HU dataset, (b) SS dataset, (c) IP dataset, and (d) KSC dataset.

The loss comparison for proposed and existing algorithms for the four datasets are shown in Figure 9. Among all the techniques, our proposed CSDCNN-AO has shown a lower loss value than other algorithms. The four efficient existing algorithms that are taken for comparison are CSDCNN, SDCNN, DCNN, and RNN. However, the loss shown in all these datasets are found to be much less than that in other existing algorithms. Especially for the KSC dataset, obtained losses are very low compared to another one. This is because the proposed technique has enhanced the effectiveness of the classification process.

## 5. Conclusion

Compressed spatial and spectral characteristics are employed as the key perception to develop a compressed synergic deep convolution neural network with Aquila optimization (CSDCNN-AO) for efficient HSI classification in this study. This combination will reduce the wavelet concept's learning difficulty and the Aquila optimization's loss function. This Aquila optimization approach may minimize the maximum number of data features without losing their characteristic state, while using less computing time and memory. Our proposed approach is superior to existing deep learning models due to higher learning ability of our synergic deep learning model based on compressed features. While comparing with the other techniques, our proposed approach can reach the maximum level of classification. In addition, the experimental results showed that the loss function does not significantly impact classification accuracy. In addition, the outcome demonstrates that the CSDCNN-AO approach has the highest accuracy among all the four datasets. Furthermore, the performance of average accuracy, total accuracy, and Kappa coefficients is optimal when implemented on all datasets. However, the proposed technique lacks optimal performance with certain samples. In future research, this issue will be resolved using a new model.

## Figures and Tables

**Table 1:** HSI categorization for Indiana Pines (IP) dataset.

Methods	RNN	DCNN	SDCNN	CSDCNN	CSDCNN-AO

OA	46.33 ± 0.45	48.73 ± 0.89	89.36 ± 1.13	89.57 ± 0.86	93.44 ± 1.08
AA	36.20 ± 1.06	49.60 ± 3.29	88.46 ± 1.17	83.14 ± 1.11	94.44 ± 1.82
K	53.97 ± 0.58	51.04 ± 1.03	89.62 ± 2.54	89.12 ± 0.26	98.33 ± 1.25
1	22.89 ± 1.09	1.33 ± 7.33	90.00 ± 1.03	30.21 ± 30.0	93.77 ± 11.6
2	45.46 ± 5.00	41.53 ± 3.04	87.35 ± 3.80	81.79 ± 0.26	90.38 ± 4.87
3	26.69 ± 2.61	30.91 ± 8.28	87.18 ± 7.24	75.93 ± 1.26	90.06 ± 4.53
4	22.79 ± 9.7	21.17 ± 3.25	83.17 ± 5.52	89.11 ± 1.12	96.84 ± 4.89
5	37.71 ± 6.67	69.79 ± 2.13	86.75 ± 2.55	79.28 ± 1.34	95.65 ± 1.95
6	89.57 ± 1.71	91.78 ± 0.78	89.08 ± 3.06	92.82 ± 0.32	96.95 ± 0.96
7	39.54 ± 11.4	19.85 ± 7.59	69.89 ± 29.7	39.69 ± 2.13	91.48 ± 24.0
8	87.46 ± 2.15	87.84 ± 3.15	85.25 ± 2.40	99.22 ± 0.31	89.11 ± 3.09
9	47.78 ± 19.04	0.00 ± 0.00	49.0 ± 49.0	19.00 ± 2.05	92.72 ± 8.38
10	49.46 ± 1.91	52.53 ± 1.24	86.47 ± 7.69	74.28 ± 0.89	92.40 ± 2.86
11	70.89 ± 2.49	61.88 ± 4.33	91.88 ± 5.03	91.12 ± 0.25	93.97 ± 3.29
12	37.14 ± 5.56	37.46 ± 3.85	77.82 ± 5.16	85.88 ± 2.37	87.56 ± 3.44
13	32.68 ± 7.17	85.02 ± 1.22	96.26 ± 5.29	50.86 ± 3.56	98.89 ± 0.87
14	81.32 ± 8.95	89.94 ± 2.59	89.16 ± 2.22	93.89 ± 1.37	96.89 ± 2.57
15	45.75 ± 5.12	44.64 ± 4.56	94.00 ± 7.49	93.76 ± 1.98	89.74 ± 2.65
16	29.60 ± 34.12	95.38 ± 1.94	99.89 ± 3.86	98.11 ± 2.67	96.89 ± 4.98

**Table 2:** HSI categorization for KSC dataset.

methods	RNN	DCNN	SDCNN	CSDCNN	CSDCNN-AO

OA	45.22 ± 0.45	47.89 ± 0.89	88.36 ± 1.13	87.48 ± 0.86	92.33 ± 1.08
AA	37.31 ± 1.06	56.60 ± 3.29	85.39 ± 1.17	82.43 ± 1.11	93.44 ± 1.82
K	52.97 ± 0.58	53.12 ± 1.03	91.62 ± 2.54	93.12 ± 0.26	97.22 ± 1.25
1	22.89 ± 1.09	30.33 ± 7.33	89.00 ± 1.03	29.21 ± 30.0	84.77 ± 11.6
2	44.46 ± 5.00	42.53 ± 3.04	86.35 ± 3.80	84.79 ± 0.26	92.38 ± 4.87
3	46.69 ± 2.61	53.91 ± 8.28	86.18 ± 7.24	85.93 ± 1.26	93.06 ± 4.53
4	29.79 ± 9.7	21.17 ± 3.25	84.17 ± 5.52	88.11 ± 1.12	95.84 ± 4.89
5	36.71 ± 6.67	68.79 ± 2.13	85.75 ± 2.55	87.28 ± 1.34	94.65 ± 1.95
6	88.57 ± 1.71	92.78 ± 0.78	88.08 ± 3.06	93.82 ± 0.32	95.95 ± 0.96
7	38.34 ± 11.4	20.85 ± 7.79	71.89 ± 29.45	42.69 ± 2.13	92.48 ± 24.0
8	91.46 ± 2.15	94.84 ± 3.15	91.25 ± 2.40	95.22 ± 0.31	97.11 ± 3.09
9	64.78 ± 19.04	0.00 ± 0.00	54.0 ± 49.0	32.00 ± 2.05	89.72 ± 8.38
10	61.46 ± 1.91	61.53 ± 1.24	89.47 ± 7.69	81.28 ± 0.89	98.40 ± 2.86
11	79.89 ± 2.49	66.88 ± 4.33	93.77 ± 5.03	95.85 ± 0.67	93.97 ± 3.29
12	47.14 ± 5.56	47.57 ± 3.85	77.97 ± 5.16	83.77 ± 4.37	95.89 ± 4.55
13	49.68 ± 7.17	87.02 ± 1.22	97.45 ± 5.29	79.86 ± 4.56	97.89 ± 0.87

**Table 3:** HSI categorization for Salinas Scene (SS) dataset.

Methods	RNN	DCNN	SDCNN	CSDCNN	CSDCNN-AO

OA	46.78 ± 1.45	57.49 ± 1.39	89.65 ± 1.13	91.89 ± 0.86	95.77 ± 1.08
AA	78.20 ± 1.06	49.60 ± 3.29	88.46 ± 1.17	83.14 ± 1.11	94.44 ± 1.82
K	53.97 ± 0.58	51.04 ± 1.03	89.62 ± 2.54	89.12 ± 0.26	98.33 ± 1.25
1	22.89 ± 1.09	1.33 ± 7.33	90.00 ± 1.03	30.21 ± 30.0	93.77 ± 11.6
2	45.46 ± 5.00	41.87 ± 3.04	88.79 ± 2.80	82.69 ± 0.26	92.56 ± 4.87
3	26.69 ± 2.61	30.91 ± 8.28	87.18 ± 7.24	75.93 ± 1.26	90.06 ± 4.53
4	22.79 ± 9.7	21.17 ± 3.25	83.17 ± 5.52	89.11 ± 1.12	96.84 ± 4.89
5	37.71 ± 6.67	69.79 ± 2.13	86.75 ± 2.55	79.28 ± 1.34	95.65 ± 1.95
6	89.57 ± 1.71	91.78 ± 0.78	89.08 ± 3.06	92.82 ± 0.32	96.95 ± 0.96
7	39.54 ± 11.4	19.85 ± 7.59	69.89 ± 29.7	39.69 ± 2.13	91.48 ± 24.0
8	87.46 ± 2.15	87.84 ± 3.15	85.25 ± 2.40	96.22 ± 0.31	88.11 ± 3.09
9	51.81 ± 19.04	0.00 ± 0.00	48.0 ± 49.0	22.00 ± 2.05	95.72 ± 8.38
10	67.46 ± 1.91	63.53 ± 1.24	90.47 ± 6.87	87.28 ± 0.89	95.30 ± 2.86
11	90.56 ± 3.49	73.88 ± 4.33	94.57 ± 5.03	95.21 ± 0.23	97.94 ± 3.29
12	54.14 ± 5.56	38.46 ± 3.85	76.47 ± 5.16	92.77 ± 3.37	92.56 ± 3.44
13	34.47 ± 7.17	83.02 ± 1.22	97.26 ± 5.29	49.93 ± 3.56	99.89 ± 0.87
14	84.32 ± 8.95	87.94 ± 2.59	99.16 ± 2.22	92.89 ± 1.37	95.89 ± 2.57
15	48.75 ± 5.12	47.64 ± 4.56	91.00 ± 7.49	96.76 ± 2.98	93.74 ± 2.65
16	34.60 ± 34.12	97.38 ± 1.94	97.89 ± 3.86	99.11 ± 2.67	95.89 ± 4.98

**Table 4:** HSI categorization for the Houston U dataset.

Methods	RNN	DCNN	SDCNN	CSDCNN	CSDCNN-AO

OA	49.23 ± 1.45	57.49 ± 1.39	88.73 ± 1.13	93.78 ± 0.86	94.67 ± 1.08
AA	78.20 ± 1.06	49.60 ± 3.29	88.46 ± 1.17	83.14 ± 1.11	94.44 ± 1.82
K	53.97 ± 0.58	51.04 ± 1.03	89.62 ± 2.54	89.13 ± 0.26	97.33 ± 1.25
1	27.89 ± 1.09	1.66 ± 7.33	92.00 ± 1.03	37.21 ± 30.0	94.77 ± 11.6
2	49.46 ± 5.00	42.87 ± 3.04	89.79 ± 2.80	82.69 ± 0.26	92.56 ± 4.87
3	26.69 ± 2.61	30.91 ± 8.28	87.18 ± 7.24	75.93 ± 1.26	90.06 ± 4.53
4	22.79 ± 9.7	21.17 ± 3.25	83.17 ± 5.52	89.11 ± 1.12	96.84 ± 4.89
5	37.71 ± 6.67	69.79 ± 2.13	86.75 ± 2.55	79.28 ± 1.34	95.65 ± 1.95
6	88.79 ± 1.71	92.78 ± 0.78	88.08 ± 3.06	93.82 ± 0.32	98.95 ± 0.96
7	38.54 ± 11.4	20.85 ± 7.59	72.89 ± 29.7	40.69 ± 2.13	92.48 ± 24.0
8	89.96 ± 2.15	88.84 ± 3.15	84.25 ± 2.40	99.33 ± 0.31	86.11 ± 3.09
9	57.81 ± 19.04	0.00 ± 0.00	76.0 ± 49.0	28.00 ± 2.05	99.72 ± 8.38
10	67.46 ± 1.91	67.53 ± 1.24	90.47 ± 6.87	87.28 ± 0.89	95.30 ± 2.86
11	82.56 ± 3.49	73.88 ± 4.33	94.57 ± 5.03	95.21 ± 0.23	99.84 ± 3.29
12	54.14 ± 5.56	48.46 ± 3.85	77.47 ± 5.16	92.77 ± 3.37	92.56 ± 3.44
13	34.47 ± 7.17	83.02 ± 1.22	97.26 ± 5.29	49.93 ± 3.56	97.89 ± 0.87
14	84.32 ± 8.95	93.94 ± 2.59	89.16 ± 2.22	95.89 ± 1.37	95.89 ± 2.57
15	48.75 ± 5.12	47.64 ± 4.56	98.00 ± 7.49	96.77 ± 2.98	93.74 ± 2.65

## Data Availability

The dataset can be obtained from the corresponding author based on the request.
